# C-reactive protein dissociation drives choroidal neovascularization in age-related macular degeneration

**DOI:** 10.1038/s41598-025-16631-z

**Published:** 2025-08-26

**Authors:** Maria Hernandez, Sara Romero-Vázquez, Sergio Recalde, Jaione Bezunartea, Maite Moreno Orduña, Idoia Belza-Zuazu, Alfredo García-Layana, Alfredo Adán, Patricia Fernández-Robredo, Blanca Molins

**Affiliations:** 1 Retinal Pathologies and New Therapies Group, Experimental Ophthalmology Laboratory, Department of Ophthalmology, Clínica Universidad de Navarra, Pamplona, Spain; 2 Navarra Institute for Health Research, IdiSNA, Pamplona, Spain; 3 Institut d’Investigacions Biomèdiques Agustí Pi i Sunyer (IDIBAPS), Ocular Inflammation Group, Barcelona, Spain; 4 Instituto Clinico de Oftalmologia, Hospital Clínic Barcelona, Barcelona, Spain

**Keywords:** Age macular degeneration, Choroidal neovascularization, Inflammation, Retinal pigment epithelium, Complement pathway, C-reactive protein, Cell biology, Immunology

## Abstract

Choroidal neovascularization (CNV) and inflammation play an important role in retinal disease development and the acute phase reactant C-reactive protein (CRP) has been shown to contribute to Age-related macular degeneration (AMD) in vitro. Our aim was to evaluate whether monomeric and pentameric CRP (pCRP, mCRP) isoforms contribute to CNV in vivo and to characterize the mechanism of CRP dissociation in-vivo and in vitro. Both CRP isoforms were intravitreally (IVT) or intravenously (IV) injected in mice, CNV was laser-induced, retinography and fluorescein angiography were performed to evaluate edema. Lectin, mCRP, F4/80 and C5b9 localization were assessed by immunofluorescence and visualized under a confocal microscope. CNV, intensity of fluorescence of mCRP (IF mCRP) was also quantified. To confirm pCRP dissociation in RPE cells and mice, pCRP was coupled to a fluorochrome and IVT injected. A statistical increase in CNV areas was observed in pCRP IVT injected males (*p* < 0.05) while a statistical decrease was shown in females (*p* < 0.05). After IV injection, pCRP males showed an increase in CNV areas only vs. mCRP injected mice (*p* < 0.05) and in females the injection of pCRP injected mice showed higher CNV areas vs. vehicle (*p* < 0.05) and vs. mCRP injected mice (*p* < 0.05). Retinal edema after IVT CRP injection was observed mainly in mCRP injected mice. In females there was an IF mCRP statistical decrease in pCRP IVT injected mice vs. vehicle and a statistical increase in pCRP IV injected mice vs. vehicle (*p* < 0.05). Mice injected with IVT isoforms showed F4/80 positive cells and C5b-9 deposition around the CNV areas. mCRP labeling was observed in the intercellular space of the endothelial cells in the angiogenic area and detected in pCRP IVT injected animals, demonstrating the dissociation of pCRP into mCRP both in vitro and in vivo in proinflammatory microenvironments. In conclusion, CRP administration increased the area of CNV and the edema observed in the subretinal space, suggesting that CRP is activated in the CNV inflammatory environment. In addition, we demonstrated that pCRP dissociates in vivo into mCRP in damaged areas close to CNV, hypothesizing that the CNV process is exacerbated by mCRP.

**Supplementary Information:**

The online version contains supplementary material available at 10.1038/s41598-025-16631-z.

## Introduction

AMD is a multifactorial neurodegenerative disease that affects people older than 55 years in industrialized countries and causes irreversible vision loss and alters their quality of life^1^. The number of patients affected is expected to rise in the world, from around 200 million in 2020 to nearly 300 million in 2040^2^. AMD classification includes early, intermediate and a late stage that can be divided into neovascular and geographic atrophy^3,4^. Neural retina and RPE cells undergo changes during choroidal neovascularization (CNV), a key process in the development of neovascular AMD leading to photoreceptors loss, especially in the macula, the center area of the retina, responsible for the ability to discern fine details^5^. As more severe retinal degeneration occurs, the patient may experience blurred vision, distortion of straight lines, reading difficulties or a central scotoma. Local inflammation, complement system and immune-mediated processes participate in the progression of AMD^6–8^ and a link between raised levels of CRP and the risk of developing late-stage AMD has been observed^9–14^.

C-reactive protein (CRP), a prototypical acute phase reactant, is a dynamic protein undergoing conformational changes between its soluble pentameric isoform (pCRP) and its insoluble tissue-bound monomeric isoform (mCRP) upon activation in inflammatory microenvironments, thereby acquiring distinct functionality. CRP is an initiator to the classical complement pathway (CCP) through binding to a wide range of substrates and is capable of regulating the alternative complement pathway^15^.

Moreover, it is known that mCRP is key to activating the CCP via C1q, C4, C2, C3 and C5b-9^16,17^. Dissociated mCRP ( shows a distinct solubility and differential biological effects^18^ than pentameric pCRP. Indeed, mCRP but not pCRP has shown to confer a proinflammatory phenotype in different cell types, including the retinal pigment epithelium (RPE)^19^ and choroidal endothelial cells^20^. Both CRP conformations show different interactions with components of the complement cascade^15,20^. CRP does not seem to be constitutively expressed in ocular tissue, but it has been identified in RPE/choroid being mCRP the predominant conformation^21,22^. In vitro studies suggest that mCRP can dissociate in the choroid and then reach the RPE (Aging) inducing barrier disruption. Indeed, mCRP has been identified in ocular drusen, a clinical early sign in AMD, with complement factors (C3a and C5a) and matrix metalloproteinases^23,24^. Extracellular deposits of lipids and cellular debris which are found within the layers of the retina and other subepithelial deposits as well as in the choroid^25–27^. Complement Factor H (CFH) has been shown to bind mCRP rather than pCRP and dampen the proinflammatory effects of mCRP^17^. Notably, CFH from patients carrying the Y402H risk variant is unable to efficiently bind mCRP and therefore the proinflammatory effects of mCRP remain unrestrained. In this regard, eyes homozygous for the high-risk CFH genotype present increased levels of mCRP within the choriocapillaris and Bruch’s membrane^21^ and increased mCRP leads also to increased granulocyte colony stimulating factor (G-CSF) in RPE/choroid explants^28^.

The aim of this study was to evaluate whether CRP isoforms contribute to CNV in vivo using the mouse model of laser-induced CNV. Given that mCRP represents the tissue-bound insoluble conformation whereas pCRP is the circulating soluble isoform, we aimed to evaluate the impact of both systemic (intravitreous, IV) and local (intravitreal) injection of CRP isoforms on CNV. We also aimed to characterize CRP dissociation both in vitro and in vivo and using multicolor immunofluorescence confocal microscopy we characterized the CNV microenvironment induced by mCRP. The understanding of CRP dynamics in retinal damage like CNV, could help to a better understanding of the contribution of CRP in AMD and the promising advancements in AMD clinical trials and therapies.

## Results

### Effect of IVT and IV injection of CRP isoforms on CNV-Lectin area

**Fig. 1:**
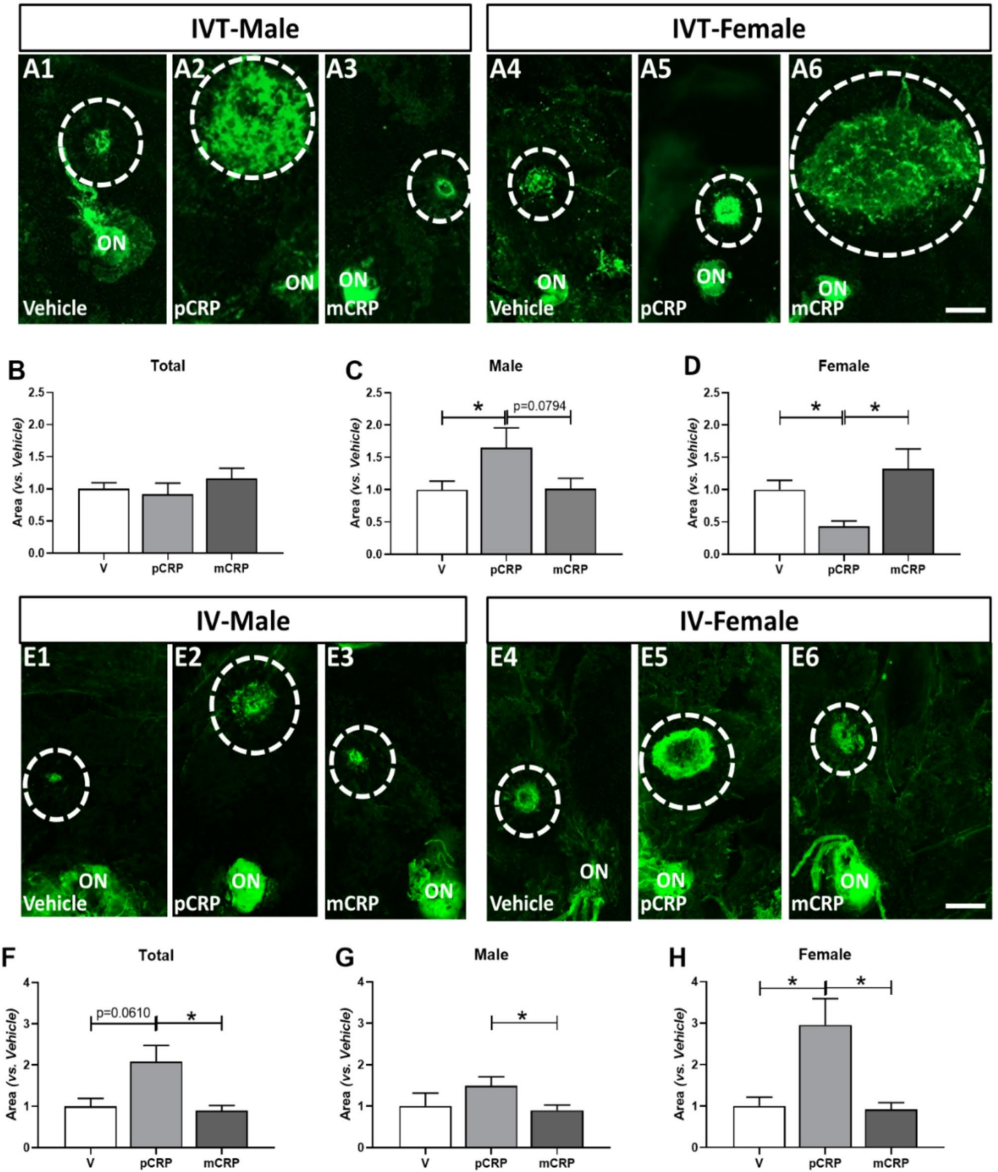
Quantification of CNV areas after IVT (sacrificed at 11 days postlaser) and IV (sacrificed at 9 days postlaser) mice injected with CRP isoforms. Analysis of the total area of CNV lectin staining in IVT mice (**A**,**B**), in males (A1-A3, **C**) and females (A4-A6, **D**). pCRP injected group showed a statistically significant increase in CNV areas vs. vehicle in males (*p < 0.05, **C**), and females showed an increase in mCRP injected group vs. vehicle and vs. pCRP (*p < 0.05, **D**). The results in IV injected mice showed a statistically significant total increase area of CNV in pCRP injected group vs. mCRP group (*p < 0.05, **F**). In males this increase is similar in pCRP injected mice vs. mCRP (*p < 0.05, E1-E3, **G**) and in females vs. V (*p < 0.05) and mCRP injected mice (*p < 0.05, E4-E6, **H**). IVT mice were injected with CRP isoforms after 1 day postlaser and IV mice were injected 1 day before and 2 days after performing the laser. Dotted circles indicate an example of the representative CNV area stained with lectin. Abbreviations: choroidal neovascularization = CNV, intravitreal = IVT, intravenous = IV, V = vehicle, mCRP = monomeric CRP, pCRP = pentameric CRPand optic nerve = ON. Scale bar: 200 μm.

To assess the effect of CRP isoforms on laser-induced CNV in males and females, we quantified the area of CNV delimited by lectin staining in injected pCRP and mCRP vs. vehicle group, respectively after IVT (Fig. 1A-F) or IV injection (Fig. 1E–H). There was non-statistically significant increase in total lesion size in mice injected with mCRP vs. V after IVT injection (Fig. 1B). This result varies depending on gender, and a statistically significant increase in CNV areas was observed in males injected with pCRP vs. vehicle (*p < 0.05) (Fig. 1A1, A2, A3, C) and almost significant vs. mCRP injected group (*p* = 0.0794). However, in females, we observed an increase in CNV areas in mCRP injected vs. pCRP and a decrease of the area in mice injected with pCRP vs. vehicle (*p < 0.05; Fig. 1A4, A5, A6, F). After IV injection of CRP isoforms, we observed a significant total area increase in pCRP injected mice vs. mCRP (*p < 0.05) (Fig. 1F) and almost significant vs. vehicle (*p* = 0.06, Fig. 1F). Analysis in pCRP injected males showed a significant increase in lectin areas vs. mCRP (*p < 0.05, Fig. 1E1, E2, E3, G) and in females this increase was statistically significant vs. vehicle and vs. mCRP (*p < 0.05, Fig. 1E4, E5, E6, H).

### IVT injection of mCRP induces edema after CNV induction

**Fig. 2:**
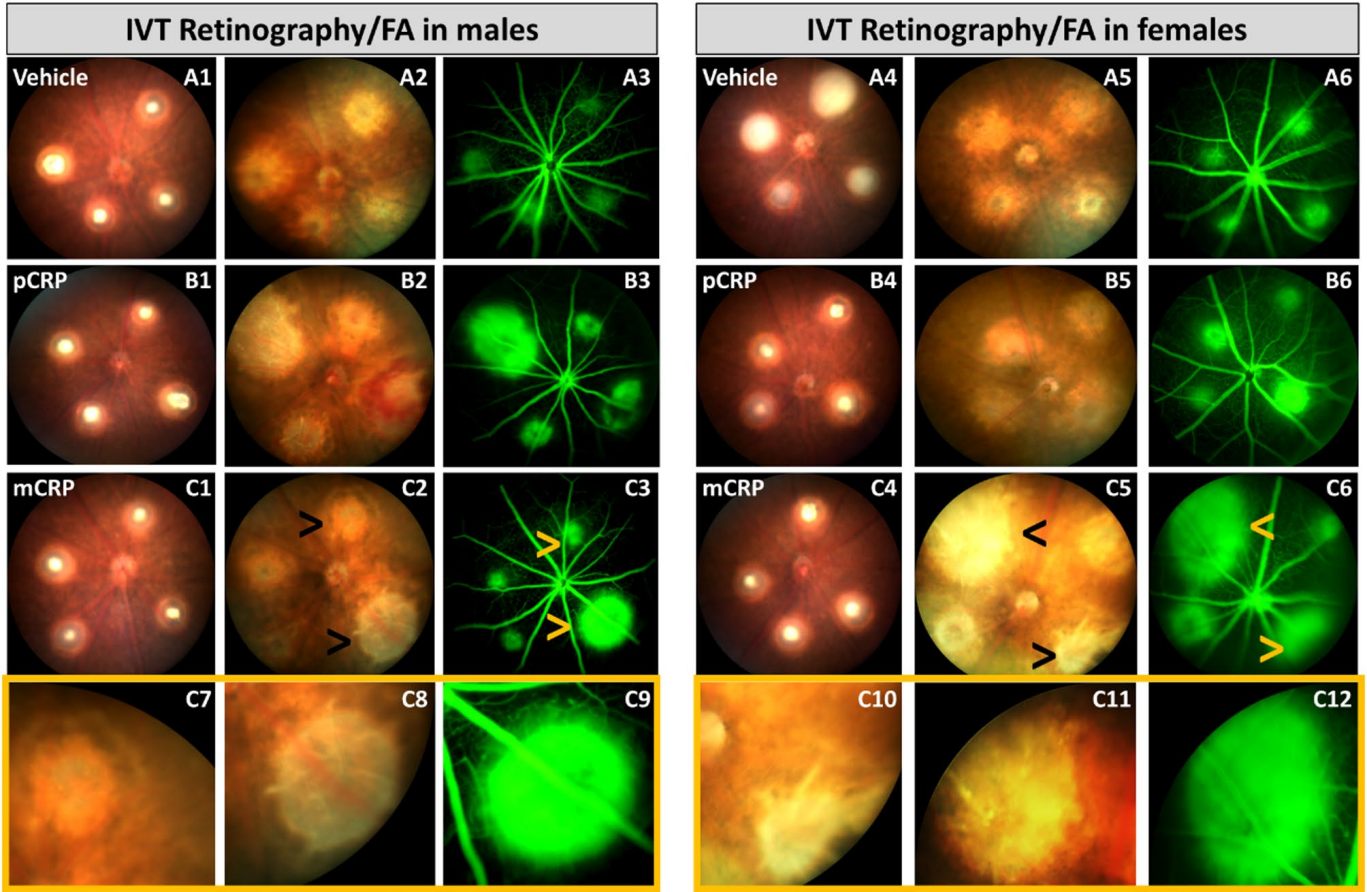
Multimodal imaging of eye fundus in mice IVT injected (sacrificed at 11 days postlaser). Retinography immediately after laser induction in males (**A1**,**B1**,**C1**) and females (**A4**,**B4**,**C4**). Retinography after 3 days from laser in males (**A2**,**B2**,**C2**) and females (**A5**,**B5**,**C5**). Fluorescein angiography (FA) was performed after 3 days from laser in males (**A3**,**B3**,**C3**) and females (**A6**,**B6**,**C6**). Retinographies (in yellow frame) (**C7**) and (**C8**) showed edema in detail in males after IVT injection of mCRP (black and yellow arrows) and retinographies images (**C10**) and (**C11**) showed edemas in detail in females after mCRP injection (black and yellow arrows). FA (in yellow frame) (**C9**) showed edema after fluorescein injection in males injected with mCRP and FA images (**C12**) showed edema after fluorescein injection in females injected with mCRP IVT. IVT mice were injected with CRP isoforms after 1 day postlaser. Abbreviations: intravitreal = IVT, intravenous = IV, monomeric CRP = mCRP, pentameric CRP = pCRP. Asterisk showed the group statistically significant in the quantification of CNV-lectin areas (Fig. 1).

A semi-quantitative fundus evaluation showed retinal severe edema (leakage) formation in CNV lesions mainly in IVT injected mice (Fig. 2A–C) after mCRP injection (Fig. 2C1–14). In this group, females (Fig. 2C4, C5, C6) showed a higher edema in most of the lesions than males (Fig. 2C1, C2, C3). We observed this leakage in more detail in retinographies and angiographies, respectively (Fig. 2C7–C10, C11-C14). In general, no edema was observed in IV injected groups (Fig. S1A, B, C) and no differences were observed between males and females for the above parameters (data not shown). In some cases (Fig. 2B1-B3), the increase of the CNV areas quantified with lectin staining (see Fig. 1) showed higher areas in retinographies and angiographies, such as in males injected with pCRP in the IVT group (Fig. 2B1–B3). To better describe the effect of IV injection of CRP isoforms, ZO-1 immunolabeling was performed and we observed that changes in RPE cells around CNV areas were only attributed to laser impacts (Fig. S2) and this alteration was similar in vehicle, pCRP (Fig. S2C-H) and mCRP IV injected mice (Fig. S2G-J in males and females).

### Monomeric CRP Immunolabeling in mice injected with CRP isoforms

**Fig. 3:**
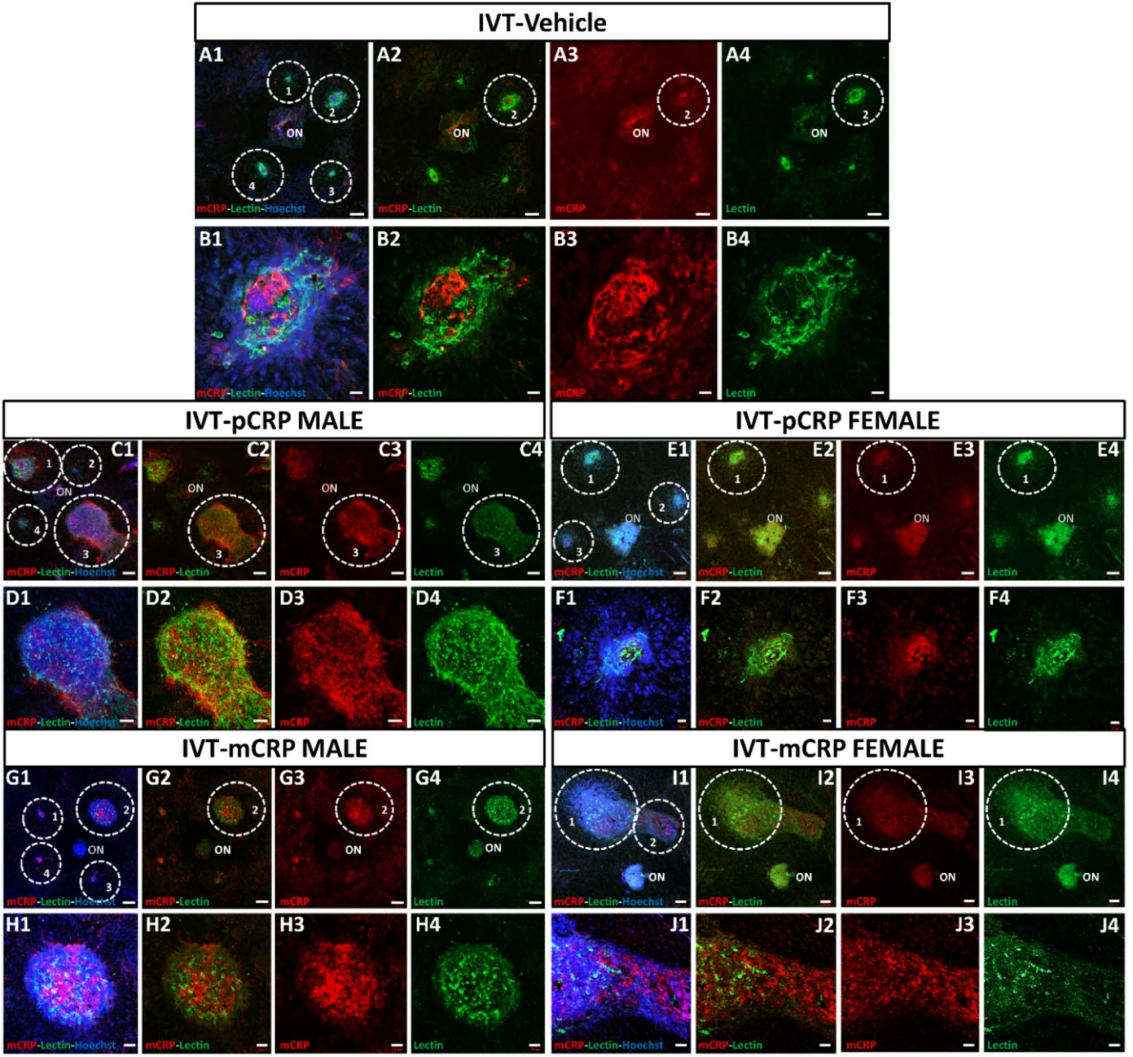
Immunofluorescence of mCRP and lectin staining in CNV areas after IVT injection (sacrificed at 11 days postlaser). RPE-flatmount stained with lectin, in green, indicates the CNV areas and mCRP labeling is shown in red. RPE-flatmounts after vehicle injection in four areas performed (**A1**–**A4**) and in detail in CNV area number 2 (**B1**–**B4**). A total mCRP immunofluorescence in mice injected with vehicle is similar to the two isoforms. In males (**C**,**D**,**G**,**H**) we observed a similar immunolabelling in four areas of CNV injected with pCRP (**C1**–**C4**) and in detail in CNV area 3 (**D1**–**D4**) vs. vehicle and vs. mCRP injected in four areas (**G1**–**G4**) and in detail in CNV area 2 (**H1**–**H4**). In females injected with pCRP (**E**,**F**) there was a decrease in labelling mCRP in four areas (**E1**,**E4**) and in detail in CNV area 1 (**F1**–**F4**) vs. pCRP injected mice in four areas (**I1**–**I4**) and in detail in CNV area 1 (**J1**–**J4**). IVT mice were injected with CRP isoforms after 1 day postlaser. The outlined regions indicate the areas of CNV around the optic nerve (ON). Hoechst (blue) label nuclei. Scale bar: 200 μm (**A1-4**, **C1-4**, **G1-4**, 100 μm (**D1-4**, **E1-4**, **I1-4**), 50 μm (**H1-4**, **J1-4**), 20 μm (**B1-4**, **F1-4**). Abbreviations: choroidal neovascularization = CNV, intravitreal = IVT, optic nerve = ON, monomeric CRP = mCRP and pentameric CRP = pCRP.

**Fig. 4:**
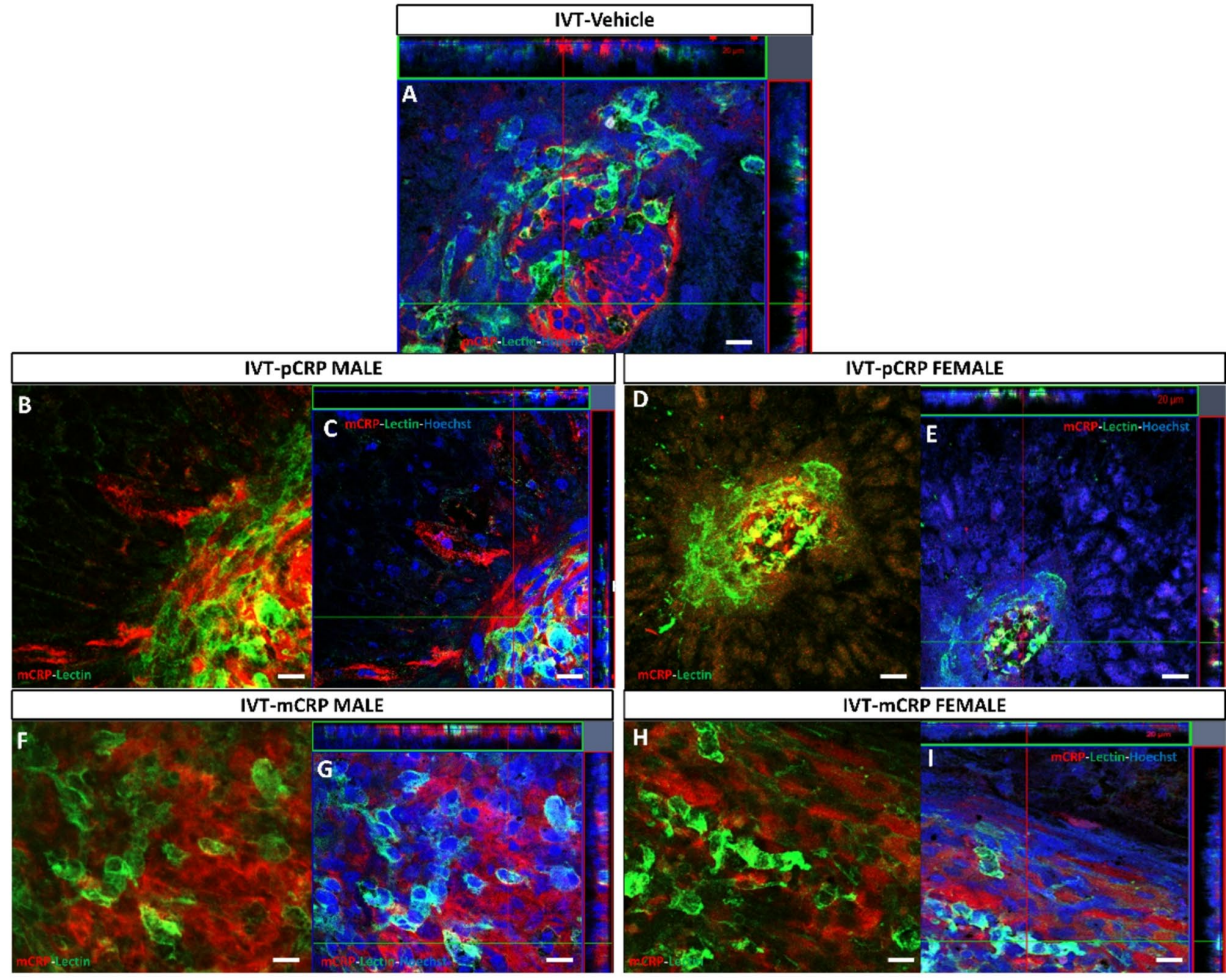
Representative higher magnification images of immunofluorescence of mCRP and lectin staining in CNV areas after IVT injection (sacrificed at 11 days postlaser). We observed in detail a specific localization of the mCRP immunofluorescence (red) that did not colocalize with endothelial cells labelled with lectin (green) in vehicles (**A**). After pCRP injection (**B**,**C**) and mCRP injection (**F**,**G**) in males, and females respectively (**D**,**E**) and images (**H**,**I**), we observed at higher magnification in orthogonal projection (**C**,**E** and **G**,**I**) the similar localization of the mCRP immunolabeling, between endothelial cells. IVT mice were injected with CRP isoforms after 1 day postlaser. Hoechst (blue) label nuclei. Scale bar: 20 μm. Abbreviations: intravitreal = IVT, monomeric CRP = mCRP and pentameric CRP = pCRP.

**Fig. 5:**
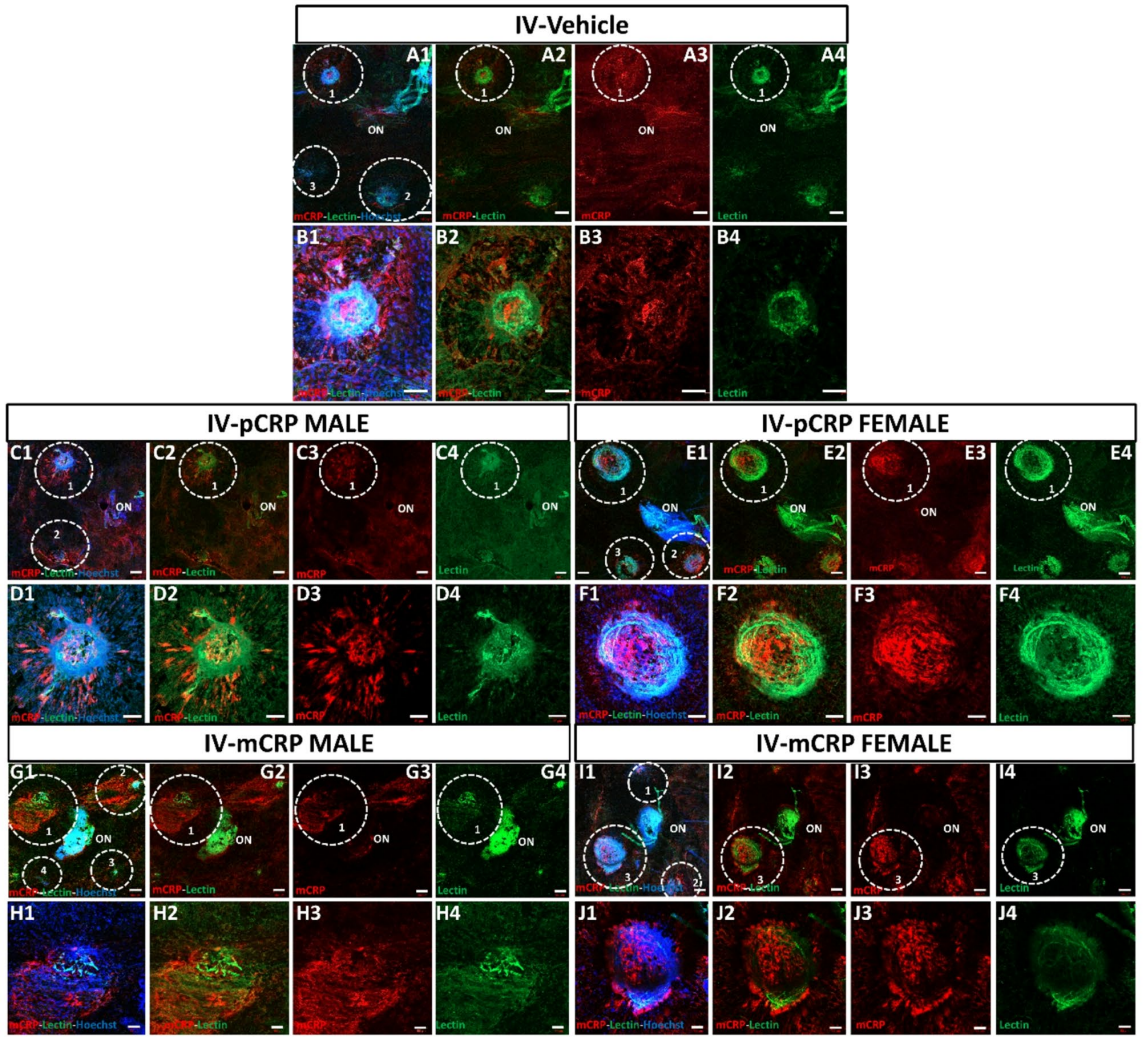
Immunofluorescence of mCRP and lectin staining in CNV areas after IV injection (sacrificed at 9 days postlaser). RPE-flatmount stained with lectin, in green, indicates the CNV areas and mCRP labeling is shown in red. RPE-flatmounts after vehicle injection in four areas (**A1**–**A4**) and in detail in CNV area number 1 (**B1**–**B4**). A total mCRP immunofluorescence in mice injected with vehicles is similar to the two isoforms. In males (**C**,**D**,**G**,**H**) we observed a similar mCRP labelling in pCRP injected mice in four areas of CNV (**C1**–**C4**) and in detail in CNV area 1 (**D1**–**D4**), and in mCRP injected mice in four areas of CNV (**G1**–**G4**) and in detail in CNV area1 (**H1**–**H4**). In females injected with pCRP (**E**,**F**) in four areas of CNV (**E1**–**E4**) and in detail in CNV area 1 (**F1**–**F4**), we showed a statistical increase of mCRP labelling in pCRP injected mice vs. and vs. mCRP injected mice in four areas of CNV (I1-I4) and in detail in CNV area 3 (**J1**–**J4**). The outlined regions indicate the areas of CNV around the optic nerve (ON). IV mice were injected 1 day before and 2 days after performing the laser. Hoechst (blue) label nuclei. Scale bar: 100 μm (**A1–4**, **C1–4**, **E1–4**, **G1–4**, **I1–4**), 50 μm (**B1–4**, **D1–4**, **F1–4**, **H1–4**, **J1–4**). Abbreviations: Choroidal neovascularization = CNV, intravenous = IV, optic nerve = ON, monomeric CRP = mCRP and pentameric CRP = pCRP.

**Fig. 6:**
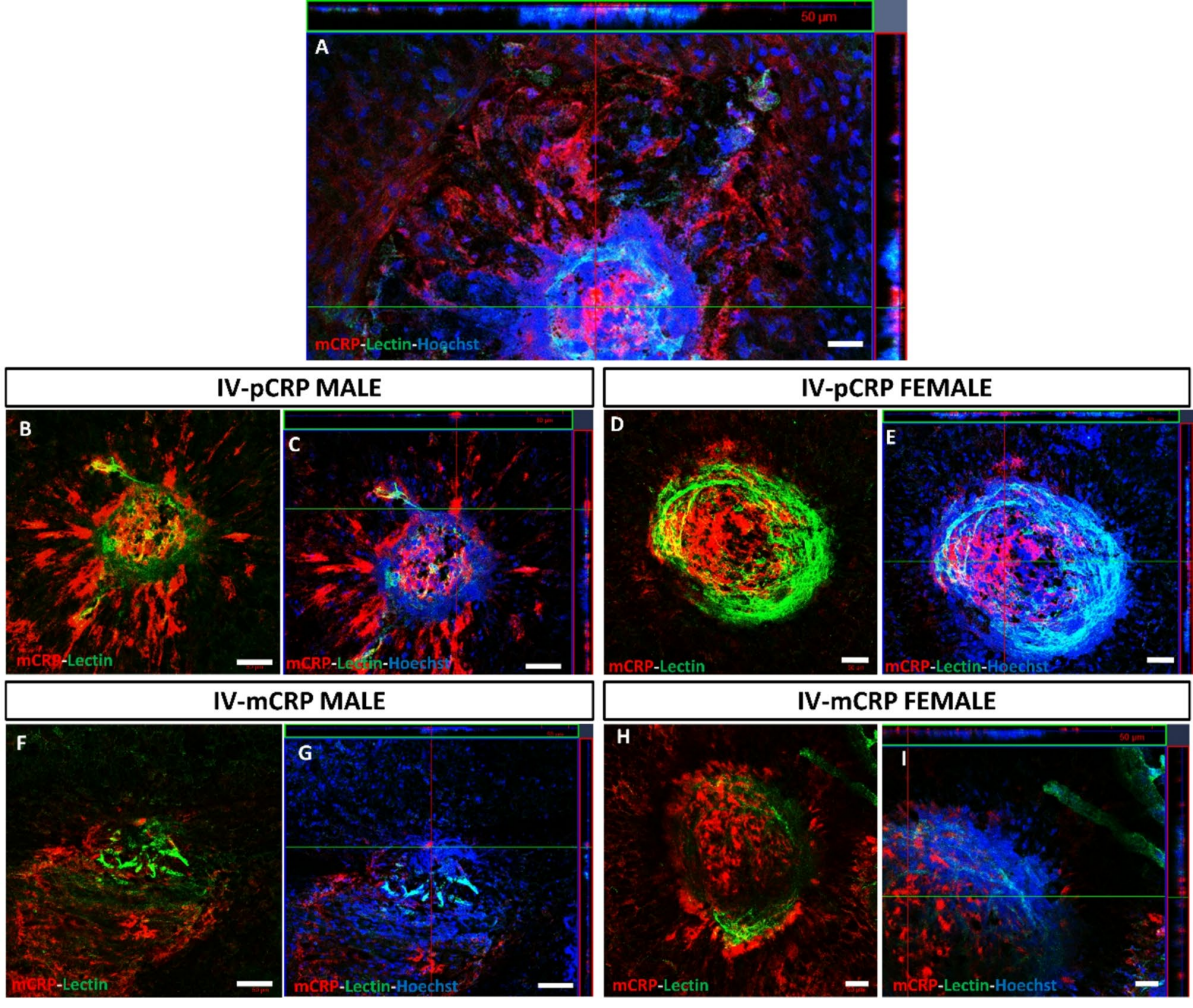
Magnification images of immunofluorescence of mCRP and lectin staining in CNV areas after IV injection (sacrificed at 9 days postlaser). The results were similar to IVT injection of the two CRP isoforms. We observed in detail a specific localization of the mCRP labelling (red) that did not colocalize with endothelial cells labelled with lectin (green) in vehicles (**A**). After pCRP injection (**B**,**C**) and mCRP injection (**F**,**G**) in males, and females respectively (**D**,**E**) and (**H**,**I**), we observed at higher magnification in orthogonal projection (**C**,**G** and **E**,**I**) the similar localization of the mCRP immunolabeling, between endothelial cells. IV mice were injected 1 day before and 2 days after performing the laser. Hoechst (blue) label nuclei. Scale bar: 20 μm (**A**), 50 μm (**B**–**I**). Abbreviations: intravenous = IV, monomeric CRP = mCRP and pentameric CRP = pCRP.

We analyzed whether mCRP was present in areas of angiogenesis in RPE-flatmounts after IVT (Figs. 3 and 4) and IV (Figs. 5 and 6) injection. After CNV lesion, in non-injected eyes, we observed a slight mCRP staining around lectin staining (Figure S2) similar to vehicle injections. A baseline mCRP labeling (red) was observed in the CNV areas close to lectin positive (green) in RPE-flatmounts from mice IVT injected with vehicle (Fig. 3A, B, Fig. S3A). In males, total IF mCRP per area of lesion was similar to vehicle (Fig. 3C, D,G, H, Fig. S3B) after both pCRP and mCRP injection. Instead, in females IF mCRP (red) per area of lesion was increased after mCRP injection vs. pCRP injection (Fig. 3E, F,I, J, Fig. S3C).

At higher magnifications in orthogonal projections (Fig. 4), we could observe that IF mCRP was mainly shown in the intercellular space of endothelial cells and in RPE cells near CNV areas. Of note is that mCRP and lectin staining did not colocalize as observed in Fig. 4B–I. The mCRP expression pattern observed was similar in both mCRP and pCRP IVT injected groups.

RPE-flatmounts from mice with IV CRP injections showed a similar IF mCRP (red) in pCRP, mCRP injected mice vs. vehicle in males (Fig. 5, Fig. S3D-F). No Notably, females injected with pCRP showed a significant increase in IF mCRP vs. vehicle (*p < 0.05) (Fig. 5E, F,I, J, Fig. S3F). At higher magnifications in orthogonal projections (Fig. 6), we could observe that IF mCRP was similar to IVT injection animals, in the intercellular space of endothelial cells and in RPE cells.

### pCRP dissociation in vitro and in vivo

**Fig. 7:**
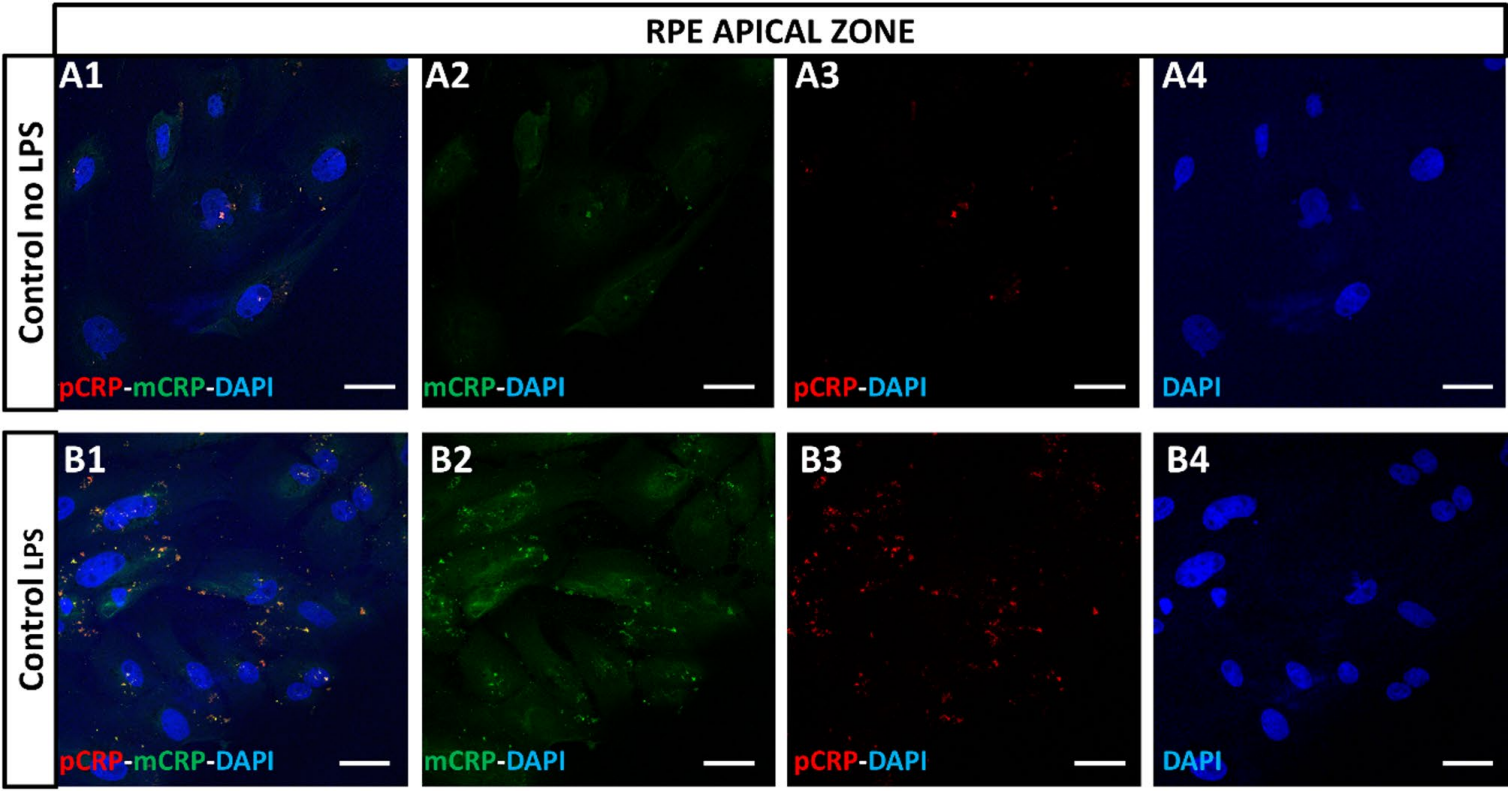
In vitro porcine RPE cells incubated with pCRP-555. RPE cells in the apical zone, control (**A1**, no LPS) and LPS incubated (**B1**) in transwell inserts were incubated with pCRP-555 (red) (**A2**,**B2**) and immunolabeled with mCRP (green) (**A3**,**B3**) and a pCRP-555 fluorochrome (red). Two isoforms were observed only in LPS-RPE cells. DAPI (blue) (**B1**–**B4**) label nuclei. Mice were injected with pCRP isoform at 1 day postlaser. Hoechst (blue) label nuclei. Scale bar: 50 μm. Abbreviations: retinal pigment epithelium = RPE, monomeric CRP = mCRP, pentameric CRP = pCRP, lipopolysaccharide = LPS.

In order to determine whether inflamed retinal tissue/RPE could induce pCRP dissociation into mCRP we performed in vitro and in vivo assays with prelabeled pCRP with a fluorochrome (HyLite 555, pCRP-555). For the in vitro assay primary RPE cells were apically treated with LPS to induce inflammation and afterwards cells were incubated with pCRP. After 48 h the presence of dissociated mCRP was evaluated by immunofluorescence in the apical zone. As observed in Fig. 7A, B, both pCRP and mCRP isoforms were observed in the cytoplasm of RPE cells whereas, in untreated cells, mCRP was not present and only little amounts of pCRP could be detected.

**Fig. 8:**
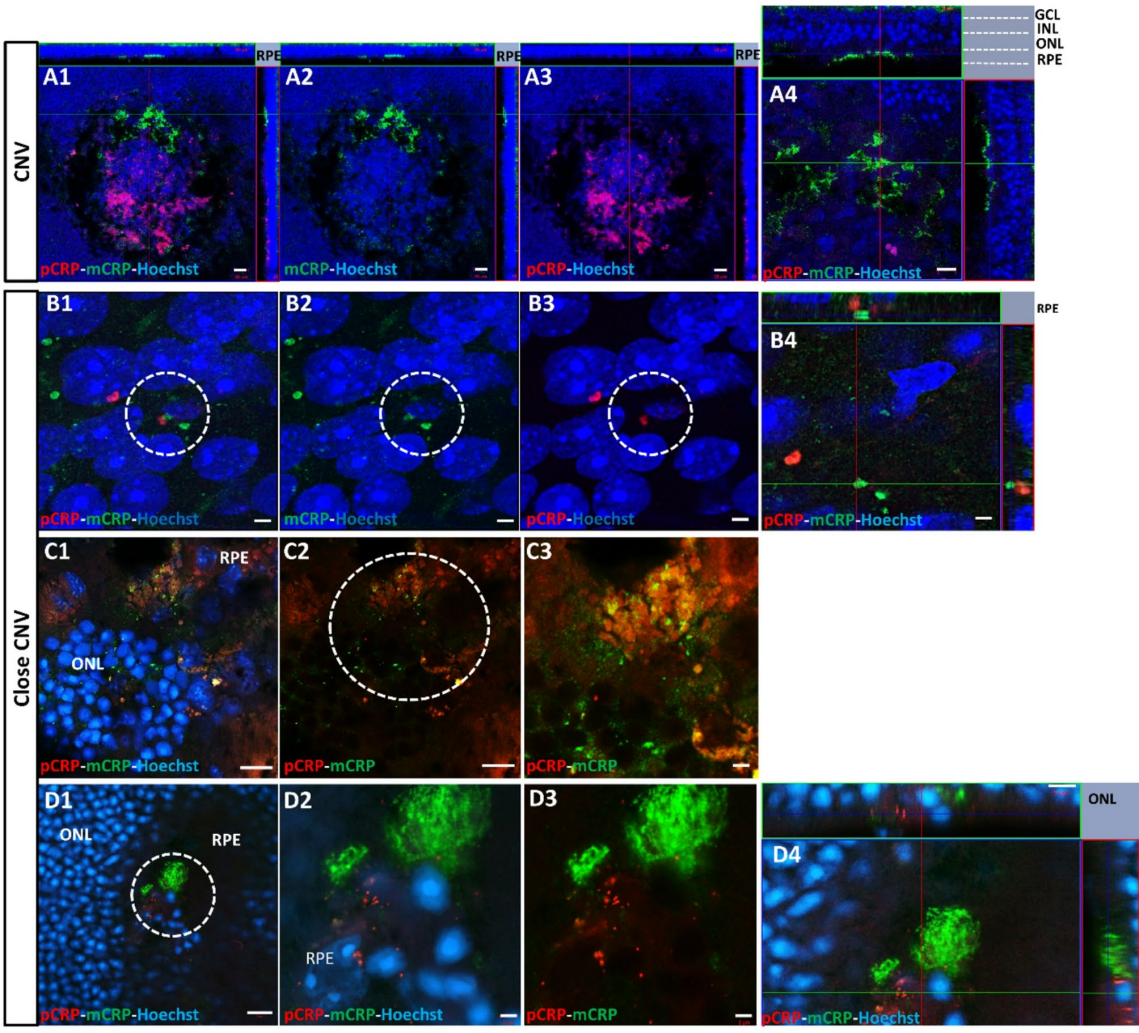
In vivo laser CNV mice model (sacrificed at 4 days postlaser) IVT injected with pCRP-555. RPE-flatmonts were immunolabeled with mCRP (green) and visualized the pCRP (red) in CNV area (**A**) and close to CNV in RPE and ONL (**B**–**D**). Super-resolution images showed the two isoforms dissociated in RPE (**B1**–**B4**) and in RPE-ONL layer (**C1**–**C3**, **D1**–**D4**). No colocalization between the two isoforms was observed in orthogonal projection (**A4**,**B4**,**D4**). In figure (**A4**) we showed the different retinal layers in orthogonal projection. The outlined regions indicate the area magnified in detail. 20 μm (A1-3), 10 μm (**A4**,**C1**,**C2**,**D1**), 5 μm (**D4**), 2 μm (**B1**–**4**,**D2**,**D3**,**C2**). Abbreviations: choroidal neovascularization = CNV, retinal pigment epithelium = RPE, intravitreal = IVT, monomeric CRP = mCRP, pentameric CRP = pCRP, outer nuclear layer = ONL, inner nuclear layer = INL, ganglion cell layer = GCL.

Dissociation of pCRP into mCRP was also observed in vivo in CNV experiments in mice, after pCRP-555 IVT injection. Both pCRP (red) and mCRP (green) were observed in CNV areas (Fig. 8A4-B4, orthogonal projections). Using super resolution, we observed in detail the two CRP isoforms in RPE cells (Fig. 8B1–B4). We examined CRP dissociation in retinal layers close to CNV areas in super resolution images and we showed the presence of the two CRP isoforms in RPE cells and outer nuclear layer (ONL) in orthogonal projection at super-resolution (Fig. 8C, D).

### Microenvironment of CNV areas after intravitreal CRP injection

**Fig. 9:**
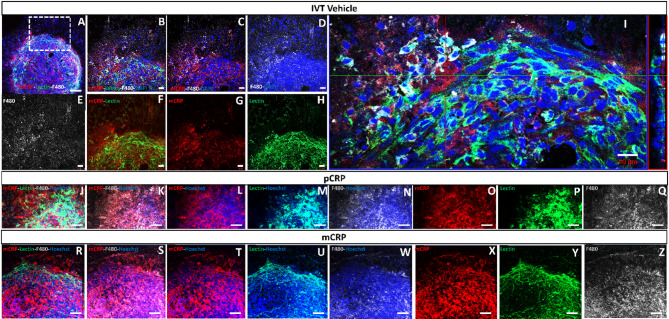
Characterization of mCRP and F4/80 immunofluorescence in a representative lectin-CNV area after IVT injection (sacrificed at 10 days postlaser). In RPE-flatmounts an increase of mCRP (red) and F4/80 (white) labeling was observed in mice injected with vehicle (**A**–**I**) as we observed in an example of a CNV area in detail in border of the CNV area with the different markers (**B**–**H**) and in orthogonal projection (**I**). After pCRP (**J**–**Q**) and mCRP (**R**–**Z**) injection in mice, increase in mCRP labeling (**O**,**X**) and a strong F4/80 immunolabeling (**Q**,**Z**) were observed compared to vehicles (**F** and **G** respectively). Mice were injected with pCRP-555 isoform after 1-day postlaser. The outlined region in image A was shown in detail in **B**–**H** images. DAPI (blue) label nuclei. Scale bar: 50 μm (**A**,**J**–**Z**), 20 μm (**E**–**I**). Abbreviations: choroidal neovascularization = CNV, intravitreal = IVT, monomeric CRP = mCRP and pentameric CRP = pCRP.

We analyzed the expression and localization of mCRP (red), F4/80 (white) and lectin staining (Fig. 9) in CNV areas after IVT injected vehicle (Fig. 9A–I), pCRP (Fig. 9J–Q) and mCRP injection (Fig. 9R–Z). Immunostaining for F4/80 confirmed that macrophages were present in areas strongly stained with mCRP (Fig. 9I) mainly in pCRP and mCRP injected mice vs. vehicle, with no evident differences between both isoforms.

### C5b9 complement complex is close to mCRP in CNV areas after intravitreal pRP injection

**Fig. 10:**
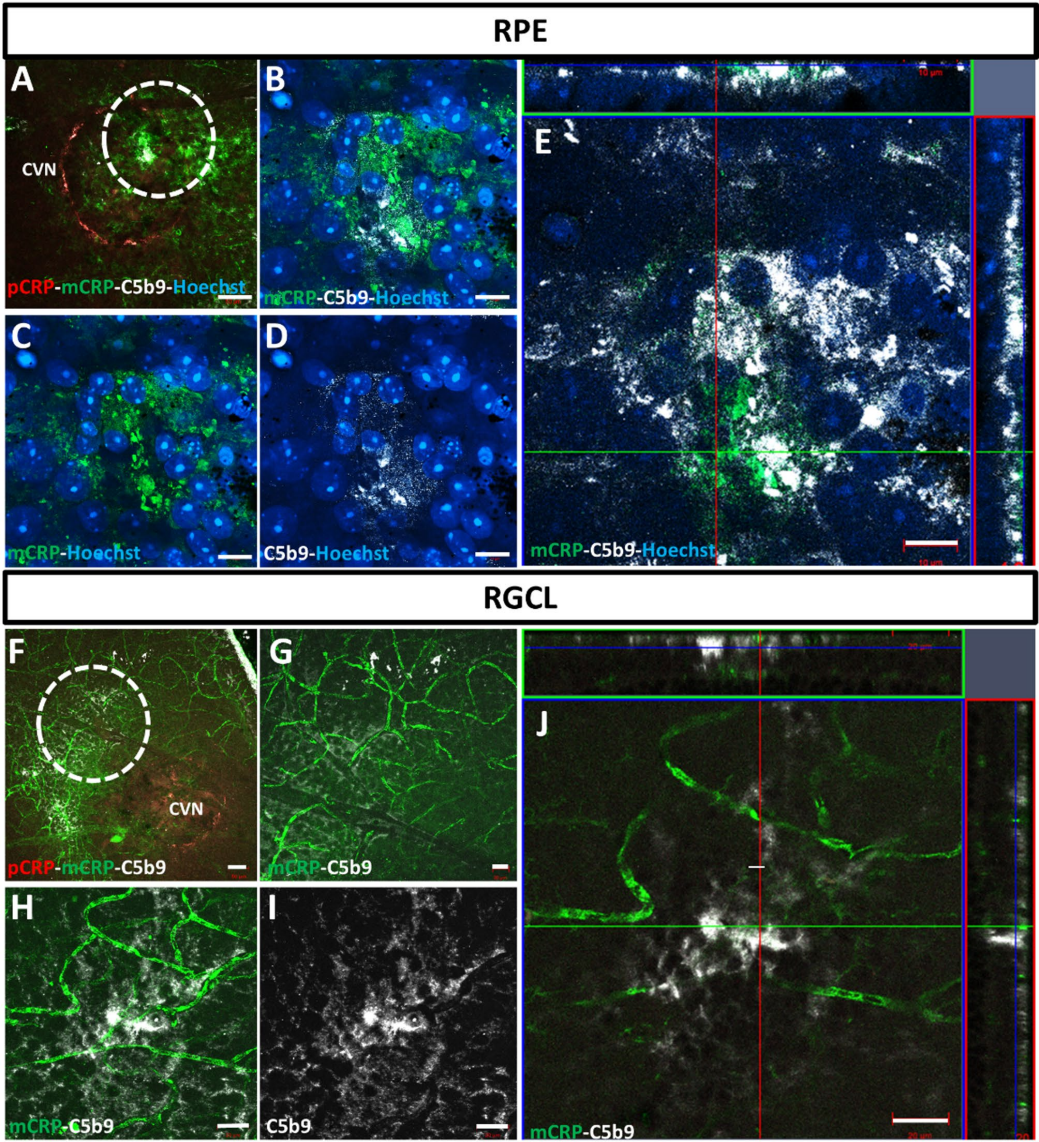
C5b9 immunofluorescence images in CNV areas (sacrificed at 4 days postlaser) in mice injected with IVT pCRP-555. In RPE layer, a light pCRP (red) labelling was observed in CNV areas (**A**) and mCRP (green) was close to C5b9 (white) (**B**–**D**), in super-resolution image and in orthogonal projection image (**E**). In retinal ganglion cell layer (RGCL) (**F**–**J**), C5b9 was observed in cells around the mCRP positive vessels (**G**) with super-resolution images (**H**,**I**) and orthogonal projection images (**J**). The mice were injected with CRP isoforms at a 1 day postlaser. Hoechst (blue) label nuclei. Scale bar: 50 μm (**A**–**D**,**F**), 10 μm (**E**) and 20 μm (**G**–**J**)). Abbreviations: choroidal neovascularization = CNV, intravitreal = IVT, monomeric CRP = mCRP, pentameric CRP = pCRP, retinal pigment epithelium = RPE, retinal ganglion cell layer = RGCL.

We observed that the complement component C5b9, was localized in RPE (Fig. 10A–G) and ganglion cell layer (GCL) (Fig. 10H–N) close to mCRP (green) in flatmounts from pCRP (red) IVT injected mice. No mCRP and C5b9 colocalization was observed in RPE cells in CNV areas in orthogonal projections (Fig. 10C, G) and in super resolution images at higher magnification (Fig. 10D–F). In retinal GCL, C5b9 positive cells (white) were found in areas close to CNV labeled with pCRP (red, Fig. 10H–K), and in vessels labeled with mCRP (green) (Fig. 10L–N).

## Discussion

This study provides, to our knowledge, the first evidence that the injection of CRP isoforms leads to an increase in CNV areas in the laser-induced CNV mouse model, with gender being a factor influencing the severity of the lesions. Moreover, IVT injection of CRP causes an increase in the edema/leakage formed in the CNV regions, with special severe effects observed in mCRP injected mice. pCRP is shown to be activated and dissociated into mCRP close to macrophages not only in the areas of angiogenesis, but also close to CNV inflammatory tissue further enhancing CNV. Indeed, we demonstrated in vitro and in vivo that pCRP dissociates into mCRP. The main results were summarized in Table 1.

**Table 1:**
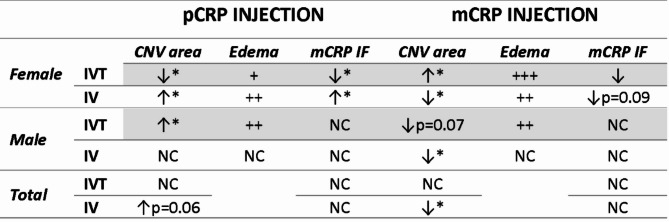
Summary of the most significant results after intravitreal (IVT) and intravenous (IV) injection of pCRP and mCRP in males, females’ mice and total data from both groups.

Arrow up indicates an increase in CNV area and fluorescence intensity of mCRP. Arrow down indicates a decrease in the CNV area and fluorescence intensity of mCRP. Semiquantitative scale is used in the presence of leakage by symbols, +: low, ++: moderate and +++: high. (*p < 0.05). Total value indicates to the sum of the data for males and females per parameter studied. Abbreviation: NC = no changes, CNV = choroidal neovascularization, IF = immunofluorescence, mCRP = monomeric C-reactive protein, pCRP = pentameric C-reactive protein.

The contribution of mCRP to AMD has been only suggested in vitro by us and others^21^ showed that mCRP induced choroidal endothelial cell migration, permeability and ICAM-1 expression^29^. They also showed evidence that mCRP is the most abundant isoform of CRP in human choroid, and that mCRP levels, in high-risk CFH genotype, were an increased tendency, suggesting a role for mCRP in the dysfunctional vascular tissues’ liability. In line with these findings, we previously showed that mCRP but not pCRP contributes to RPE barrier dysfunction and inflammation^18,30^ and that mCRP can reach the subRPE through the choroid or locally dissociate from pCRP in inflamed RPE^23^. The role of CRP has been studied previously in vivo in new vessels of atherosclerotic lesions^31,32^; however, there are no studies with regards to the effect on new vessels in CNV in the retina in vivo. We found that the effect of CRP injection on CNV areas was different depending on the isoform, the route and, interestingly, in our study, also the gender.Systemic injection (IV) of pCRP increased CNV area in females but not in males while mCRP injection had no effect and. The differences in the results could be due, among other factors, to the hormonal system of males and females, although there is not enough scientific literature about the study of the two isoforms of CRP. As seen in other publications related to systemic CRP levels in AMD patients^13,33^. female gender and CRP levels is a super-additive risk in AMD progression. In our study, it should be noted that in females, CNV area and mCRP expression in these areas, after CRP isoforms injected showed a similar pattern, being different in males, which suggest the need of integrating sex and gender in AMD studies and clinical guidelines. Also, it has been shown that females had significantly higher detection rates of systemic mCRP than males in an AMD cohort^34^. Nevertheless, the specific mechanisms that account for these differences are still not fully understood. Furthermore, these preliminary findings come from preclinical research conducted using cell cultures and animal models. On the other hand, the lack of effect of IV injected mCRP led us to hypothesize that IV mCRP does not reach the eye in a concentration able to produce the effects observed in the IVT injected groups. Given that mCRP represents the tissue-bound insoluble form of CRP it could be cleared out and/or bound to other tissue before reaching the eye. With regards to the IVT route, apparently, there was no difference in CNV areas between vehicle and CRP injections.

Besides the effect on CNV area, we observed that mCRP locally-injected (IVT) induced more edema and leakage than pCRP, suggesting that mCRP exacerbates inflammatory responses rather than new vessel development. The fact that also pCRP induced a certain level of edema suggests a partial dissociation of pCRP into mCRP and thereby the proinflammatory effect. As a matter of fact, opposite effects of CRP isoforms have been reported in endothelial progenitor cells where mCRP induces the up-regulation of proinflammatory, interferon-responsive genes whereas pCRP exhibits a primarily noninflammatory gene response^35^. We confirmed that pCRP was dissociated into mCRP in vivo after IVT injection. Further studies are needed to elucidate whether pCRP has a more proangiogenic phenotype and, once pCRP is dissociated, if mCRP can induce a proinflammatory (edema and leakage) response. Subretinally-injected CRP is suggested to induce degradation of mast cells, provoking inflammatory responses and retinal degeneration^36^. Mast cell activation and degranulation results in extracellular matrix remodeling and release of pro-angiogenic factors, leading to CNV as recently shown by other authors^37^. This effect also suggests that mCRP could increase the severity of the lesions by contributing to a sustained inflammatory environment. Although CRP is known to induce a pro-inflammatory phenotype in endothelial cells ex vivo in human coronary arteries^38^, a direct role on progression of CNV has not been established before. Unfortunately, information is scarce about the binding receptors of the two CRP isoforms. mCRP anchorage occurs mainly in membrane domains called lipid rafts^39^ when mCRPis integrated into lipid rafts, no further external receptor might be required and a signal transduction cascade involving Pi3-kinases and src-kinases takes place^39^. Specifically, it has been demonstrated that CRP may promote failure in the Na^+^/K^+^ pump in rat cerebro-microvascular endothelial cells and this event triggers the generation of edema^40^.

In addition, we observed an increase of macrophage recruitment after injection of both CRP isoforms close to CNV areas. A variety of inflammatory cell types including macrophages, lymphocytes among others are found in human subfoveal CNV membranes^41^ and macrophages participate in the inhibition of angiogenesis in AMD patients^42,43^. This observation could explain the reduction in CNV areas in cases of high edema and leakage that we found when mCRP was injected. CRP also induces pro-inflammatory cytokine release from human monocytes^44–46^, specifically IL-1β, TNF-α, IL-6^47^ and IL-10^48,49^ and studies specifically addressing the effect of CRP isoforms suggest that mCRP induces macrophage polarization to a proinflammatory M1 phenotype^50–52^. We observed in our murine experiments that macrophages were close to mCRP labeling. Although some authors have reported a certain level of expression of mCRP in U937-derived macrophages^53^, mCRP detected in the lesions most likely is the result of the deposition and dissociation of exogenously injected CRP. Eventually, murine CRP could also arrive from the systemic circulation and dissociate in the CNV areas.

The cause of CNV is still under intense investigation and inflammation and complement system appear to be important components. Individuals with the Y402H polymorphism in CFH gene show limited ability to control the alternative pathway of the complement system due to altered binding capabilities of mutant CFH to its tissue-associated ligands. Since mCRP is shown to be present in BM and choriocapillaris with C3a, C5a and matrix metalloproteinases^54^, mCRP could increase MAC deposition observed in the choriocapillaris with advanced age and disease. We have observed in angiogenic areas in RPE and in retinal ganglion cell layer, mCRP labeling with cells positive to C5b9, that did not co-localize with each other suggesting that these RPE cells probably die through MAC-mediated cell lysis. Moreover, in the retinal ganglion cell layer, mCRP and C5b9 positive cells were also observed, although we cannot claim a causal relationship, the correlational relationship suggests mCRP participation in local inflammatory processes, possibly via complement activation. However, further studies in the CNV retinal sections after pCRP injection would be necessary to determine which cell type is positive for C5b9, possibly ganglion cells, as this has been confirmed in other pathologies^55^.

Whether systemic CRP also contributes to CNV and AMD progression needs to be further studied. Our group has recently shown that mCRP/FH ratio is increased in intermediate AMD^34^ supporting a participating role of a systemic proinflammatory environment in early stages of AMD. Our experiments with systemic (IV) injection of pCRP where we observed an increase in CNV areas also support the role of systemic CRP in AMD. IV injected pCRP underwent dissociation into mCRP near inflamed CNV areas further exacerbating neovascularization. High levels of systemic pCRP are indeed a source for local mCRP in damaged/inflamed tissue. The fact that IV mCRP injection did not yield any effect in CNV suggested that mCRP may not reach the eye as it is highly insoluble and might have bound to other tissues before being able to reach the eye, thus being unable to induce any effect. Indeed, mCRP staining in animals IV injected with mCRP showed the same IF mCRP than those injected with vehicles.

To elucidate the dynamics of CRP dissociation in the eye, pCRP coupled to a fluorochrome (pCRP-Hylite 555) was IVT injected in mice one day after CNV induction. We observed the presence of both mCRP and pCRP near CNV areas showing the ability of CNV inflammatory microenvironment to dissociate pCRP into mCRP. Interestingly, although in some areas both CRP isoforms colocalized, when analyzing samples with super high-resolution, they were spatially close to each other but did not colocalize. CRP dissociation in vivo was previously shown with intravital microscopy in a LPS-induced cremasteric muscle inflammation in rats^56^. A deeper study of the mechanism and dynamics of CRP dissociation in the eye could provide insights on how to target CRP dissociation for therapeutic purposes to treat AMD.

Although robust, our study has some limitations. The CNV laser induced murine model is a validated model of CNV process observed in AMD, but it is an acute model and lacks the chronicity of the disease, therefore the translation of the results must be cautious. A chronic CNV model could be interesting to elucidate the role of CRP conformations in a long-term condition similar to AMD patients. On the other hand, the effect of mCRP on the leakage and edema in angiogenic areas would need specific technical devices such as optical coherence tomography (OCT). In addition, biodistribution data of mCRP would be necessary to conclude that systemic IV injection of mCRP does not reach the eye and data regarding serum and aqueous levels of CRP conformations post-injection would also help to better elucidate CRP dynamics in the context of AMD.

In conclusion, the present study demonstrates a dissociation of pCRP into mCRP isoform in vivo and in vitro under an inflammatory microenvironment with exacerbation of CNV in a gender-dependent manner providing evidence for the contribution of mCRP in retinal inflammatory diseases. Further studies focused on the inflammatory mechanisms after CRP injection in CNV areas of the retina and RPE/choroid are needed to understand how CRP causes changes in chronic damaged tissue and how this dissociation can be affected bygender.

## Materials and methods

### CRP isoforms

To avoid spontaneous dissociation of pCRP, high purity CRP (Abyntek) was kept in a buffer containing 0.010 M Trizma, 0.140 M NaCl, and 0.002 M CaCl2 and endotoxins were removed using the Rapid Endotoxin Removal Kit (Abcam, Cambridge, USA). mCRP was derived from pCRP by urea chelation^12^. For this purpose, pCRP (3.5 mg/mL) was chelated with EDTA 0.01 M and treated with urea 8.0 M for 3 h at 37 °C. Afterwards, dialysis against low ionic strength buffer (0.01 M Tris-HCl and 0.05 M NaCl, pH 7.3) was performed to eliminate urea. Protein concentration was measured with the BCA protein assay and the obtained mCRP solution was filtered and kept at 4 °C. Sodium azide was eliminated from the pCRP solution via dialysis against TBS. The composition of the vehicle was NaCl (0.28 M) and CaCl2 (0.005 M).

### In vitro evaluation of CRP dissociation on inflamed RPE cells

Primary porcine RPE cultures were prepared from explants isolated from fresh adult porcine eyes obtained within 3 h after slaughter from a local abattoir following the experimental protocol previously reported by Toops et al.^57^ with few changes. Connective tissue surrounding the eyes were removed and eyes were incubated in a 0.2% povidone iodine solution for 10 min on ice. Eyes were then washed with sterile distilled water and incubated with Penicillin-Streptomycin (1,000 U/mL) for 5 min at 4 °C. Once eyes were disinfected, anterior segments were eliminated at the ora serrata and vitreous and crystalline were removed. To detach the retina from the RPE monolayer, eyecups were incubated with EDTA 1mM for 30 min at 37 °C and then the retina was carefully released. Then, eyecups were incubated with 0.05% trypsin/EDTA to obtain the RPE cells. RPE cells were then seeded in DMEM High Glucose (Capricorn Scientific, Ebsdorfergrund, Germany), with L-glutamine and sodium pyruvate, supplemented with 1% penicillin-streptomycin, 1% non-essential amino acids (Corning, Life Science, Tewksbury, MA, USA) and 10% FBS. After culturing cells for three days, ciprofloxacin (5 µg/ml) was added to the culture medium and at day 7 FBS was reduced to 1% to favor RPE polarization. After two weeks RPE formed pigmented monolayers showing its specific cobblestone morphology. At this point, cells were trypsinized and seeded at high density into semi-permeable Transwell^®^ filters (Corning, Merck, Darmstadt, Germany) pore size, previously coated with laminin. Porcine RPE cells were then cultured for at least 4 weeks and fed with 1% FBS growth medium every 3–4 days.

Lipopolysaccharide (LPS) treatment was used to induce pCRP dissociation in the apical surface of RPE cells. RPE cells were treated with 100 µg/mL LPS (LPS25, Merck, Darmstadt, Germany) for 24 h before adding 50 µg/mL pCRP-HiLyte 555 (pCRP labeled with HiLyte Fluor 555 using Labeling Kit-NH2 [LK14-10, Dojindo Laboratories, Munich, Germany] following manufacturer instructions) and cells were incubated for 48 h. Afterwards, RPE cells were washed twice with PBS, fixed with 3.8% paraformaldehyde for 15 min, and washed again with PBS. Then, cells were blocked with a blocking buffer (1% BSA in PBS) for 1 h and subsequently incubated with primary monoclonal antibody anti-mCRP (CRP-8, Sigma Aldrich C1688) at 4 °C for 18 h. Samples were then washed 3x with PBS and incubated with secondary antibody Alexa Fluor anti-mouse 488 (1:200 ThermoFisher) for 1 h at RT. Nuclei were counterstained with DAPI (Sigma-Aldrich, St. Louis, MO, USA).Apical side of RPE samples were visualized on the high-speed spectral confocal microscope Leica TCS-SP5.

### Injection of CRP and induction of choroidal neovascularization using laser in mice

The study was conducted following the European Community guidelines for ethical animal care and use of laboratory animals (Directive 2010/63/UE) and approved by the University of Navarra Animal Research Review Committee (091 − 20) and the reporting in the manuscript follows the recommendations in the ARRIVE guidelines. Ten sixteen-weeks-old wild-type (WT, 5 males and 5 females) (C57Bl6/J, Charles River, Wilmington, MA, USA) mice were used for the intravenous (IV) injection study (systemic administration) and 8 sixteen-weeks-old wild-type mice (WT, 4 males and 4 females) (C57Bl6/J, Charles River, Wilmington, MA, USA, ) were used for the intravitreal (IVT) injection (*n* = 16 eyes) (local administration). Animals were housed in standard cages with a 12 h light/dark cycle with food and water ad libitum. Xylazine (10 mg/kg; Xilagesic 2%; Calier Laboratories, Barcelona, Spain) and ketamine (75 mg/kg; Imalgene 1000; Merial Laboratories, Barcelona, Spain) were used for anesthetic purposes and pupil dilation was performed by using Tropicamide (3 mg/mL; Alcon Cusí, Barcelona, Spain) and phenylephrine (7.8 mg/mL; Alcon Cusí, Barcelona, Spain). A digital laser system (532 nm; Micron IV, Phoenix Research Laboratories, OR, USA) was used to induce CNV lesions as previously published^58^. Three to four laser spots (250 mW, 0.05 s, and 50 μm size) were made close to the optic nerve. The rupture of Bruch’s membrane (BM) was confirmed by the presence of a bubble immediately after the laser application. In the case of an impact with vitreous hemorrhage or bubble absence, they were not considered for the study.

One day postlaser, pCRP, mCRP were IVT injected (1 µL, 10 µg/ml) in mice using an automated pump-based perfusion system (LEGATO^®^ 130, KD Scientific Inc. Holliston, MA, USA) and a 10 µL syringe (Hamilton Bonaduz AG, Bonaduz, Switzerland). The syringe with a 32 G needle was inserted into the vitreous in a position 1 mm behind the limbus with a 45° injection angle. Fluorescein angiography (FA) was performed at 4 days postlaser. Two animals (*n* = 4 eyes) were used to analyze mCRP immunolabelling. These animals received a vehicle in one eye and the other remained intact and were sacrificed at 1 week (Figure S2) after cervical dislocation.

**Fig. 11:**
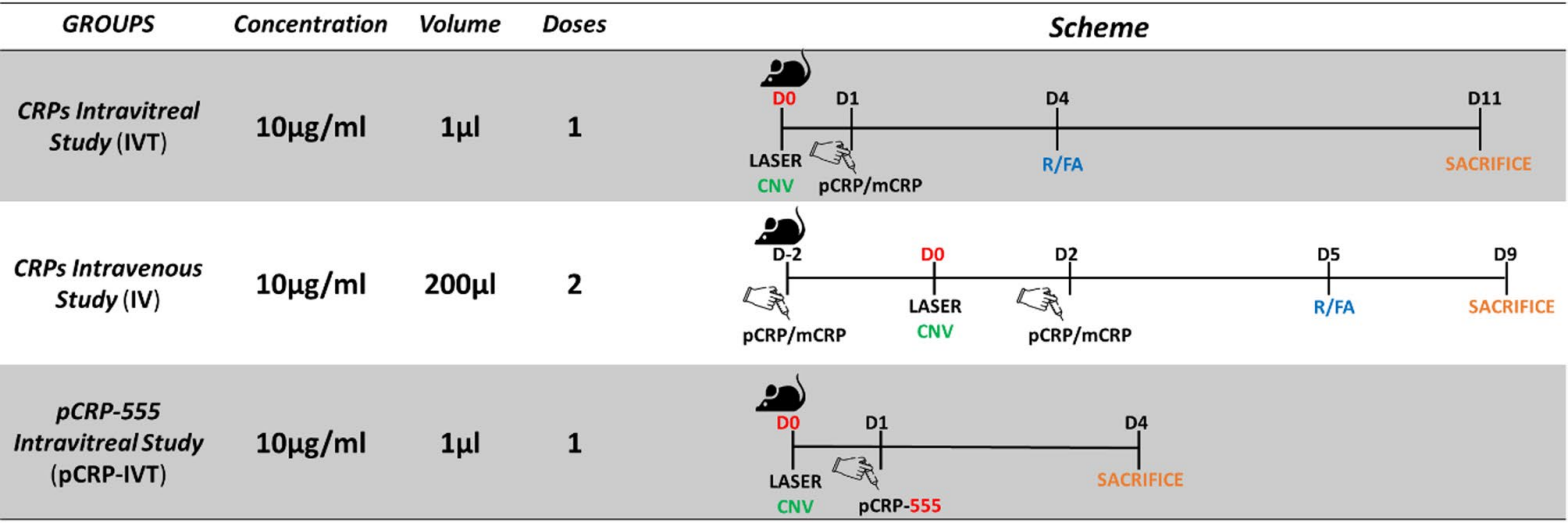
Scheme of timing and experimental procedures performed in the study with a local (intravitreal, IVT) and systemic (intravenous, IV) administration of CRP isoform. Abbreviations: monomeric C-reactive protein = mCRP, pentameric C-reactive protein = pCRP, day = D, retinography = R, fluorescein angiography = FA, choroidal neovascularization = CNV, intravitreal = IVT, intravenous = IV.

For IV injections we induced peripheral vasodilatation with local warming of the tail by a red heat lamp for 5 to 10 min prior to injection followed by ethanol (70°). Animals were placed in a restraining device in a sternal position, slightly rotating the tail to visualize the lateral vein. The needle (29G) (BD Micro-Fine, NJ, USA) was inserted parallel to the tail. IV injections (200 µL, 10 µg/ml) of pCRP, mCRP and their vehicles were performed in mice before and after CNV laser according to Fig. 11. Fluorescence angiography (FA) was performed 5 days postlaser. Six animals (3 males and 3 females) were used for IVT injection of pCRP-555 in order to observe where the pCRP was located in flat mounted RPE-retinas. HiLyte Fluor 555 Labeling Kit-NH2 (LK14-10, Dojindo Laboratories) was used for pCRP labeling following manufacturer instructions. Samples were flatmounted and images were obtained using a confocal microscope (LSM800, Zeiss, Oberkochen, Germany). Experimental schedule for IV or IVT injection of pCRP, mCRP, and IVT injection of pCRP-555 is shown in Fig. 11.

### Fluorescein angiography (FA)

At 4 days in the IVT group and 5 days in the IV group after laser application, 10% sodium fluorescein was intraperitoneally injected and early and late FA Images were captured using a digital system (Micron IV, Phoenix Research Laboratories, OR, USA). Mean leakage area (in pixels) measured using Fiji ImageJ software, (an open-source Java-based image analysis software; NIH, Bethesda, MD, USA) was calculated for each study group as described in previous studies^59^. Measurements were performed by two independent, trained, and experienced observers that were not aware of study groups.

### Choroidal flatmounts, neovascularization and Immunofluorescence

The animals were anesthetized prior to the euthanasia process using a CO2 gradient and sacrificed by cervical dislocation. Mice RPE-choroid-sclera complexes were microsurgically separated. After an incubation with blocking buffer (PBS, 3% Triton X-100, 0.5% Tween 20, 2% sodium azide, and 1% FBS) for 1 h at 4 °C, they were subjected to biotinylated isolectin, mouse monoclonal anti-mCRP antibody (clone 3H12), mouse monoclonal anti-pCRP (clone 1D6), rat polyclonal F4/80 antibody, rabbit polyclonal ZO-1 and rabbit polyclonal anti-C5b9 (Table S1) overnight at RT. Then the flatmounts were washed in PBS and incubated with the corresponding secondary antibodies and nuclei markers as detailed in Table S1: Alexa Fluor Streptavidin 488 (1:250; S32354; Life Technologies, Carlsbad, CA, USA), donkey anti-mouse 594 (1:250; A-21203, Thermo Fisher, Paisley, UK), donkey anti-rat 647 (1:250, A48272, Thermo Fisher, Paisley, UK) and donkey anti-mouse 647 (1:250, A21235, Thermo Fisher, Paisley, UK). Hoechst 33,342 (H1399, ThermoFisher, Waltham, MA, USA) and DAPI (Sigma-Aldrich, St. Louis, MO, USA) were used to label nuclei. Flatmounts were mounted with PBS-glycerol (1:1) and visualized under a confocal microscope (LSM800; Zeiss, Oberkochen, Germany). In some cases, we used super-resolution software (Zeiss Oberkochen, Germany) to capture images in confocal microscope. We quantified the intensity of fluorescence of mCRP (IF mCRP) in each lesion in RPE-flatmounts per group injected. Areas and IF were measured by Fiji ImageJ (NIH, Bethesda, MD, USA).

### Statistical analysis

Data are presented as mean ± SEM. Normal distribution of data was assessed by the Shapiro-Wilk test. One-way ANOVA followed by Bonferroni post-hoc test for multiple comparisons was used for CNV area comparisons between groups. Statistical significance was considered when *p* < 0.05. Statistical analysis and results were graphed using GraphPad Prism 8.0 (GraphPad Software, San Diego, CA, USA)^60,61^.

## Supplementary Information

Below is the link to the electronic supplementary material.

Supplementary Material 1

## Data Availability

All data generated or analysed during this study are included in this published article and its supplementary information files.

## Abbreviations

AMD: Age-related macular degeneration
BM: Bruch’s membrane
C5b-9, MAC: Membrane attack complex
CCP: Classical complement pathway
CFH: Complement factor H
CRP: C-reactive protein
CNV: Choroidal neovascularization
D: Day
DAPI: 40,6-diamidino-2-phenylindole
EDTA: Ethylene diaminetetraacetic acid
FA: Fluorescein angiography
FH: Factor H
GA: Geographic atrophy
GCL: Ganglion cell layer
LPS: Lipopolysaccharide
IF mCRP: Intensity of fluorescence of mCRP
INL: Inner nuclear layer
IV: Intravenous
IVT: Intravitreal
mCRP: Monomeric C-reactive protein
MLC: Myosin light chains
nvAMD: Neovascular AMD
ON: Optic nerve
ONL: Outer nuclear layer
pCRP: Pentameric C-reactive protein
PFA: Paraformaldehyde
R: Retinography
RGCL: Retinal ganglion cell layer
RPE: Retinal pigment epithelium
V: Vehicle
WT C57Bl6/J: Wild-type mice
