# Supplementary material for: C-reactive protein dissociation drives choroidal neovascularization in age-related macular degeneration
Source: Sci Rep. 2025 Aug 26;15:31408. doi: 10.1038/s41598-025-16631-z (PMC12381139; doi:10.1038/s41598-025-16631-z)
Supplement: Supplementary file 1 — Supplementary Material 1 [file 41598_2025_16631_MOESM1_ESM.pdf]

Supplementary material

MARKER	REFERENCE	DILUTION	LOCALIZATION
Isolectin GS-IB4 Biotinilated	Vector, B-1205	1:240	Endotelial cells
Mouse monoclonal anti-**mCRP** antibody	3H12, gently provided by Dr. LA Potempa	1:100	mCRP
Mouse monoclonal anti-**pCRP** antibody	1D6, gently provided by Dr. LA Potempa	1:100	pCRP
Rat-polyclonal **F4/80** antibody	Abcam, ab6640	1:100	Macrophages
Rabbit polyclonal **ZO-1** antibody	Abcam, ab59720	1:100	Tight junctions
Rabbit polyclonal **C5b9** antibody	Biorbyt, orb499686	1:250	C5b-9, or membrane attack complex
Alexa Fluor Streptavidin 488	Life Technologies, S32354	1:250	Secondary antibody
Donkey anti-mouse 594	Thermo Fisher, A-21203	1:250	Secondary antibody
Donkey anti-rat 647	Thermo Fisher, A48272	1:250	Secondary antibody
Donkey anti-mouse 647	Thermo Fisher, A21235	1:250	Secondary antibody
Hoescht	Thermo Fisher H1399	1:2000	Nuclei
DAPI	Biotium, 40009	1:800	Nuclei

Table S1 Antibodies used in the study.

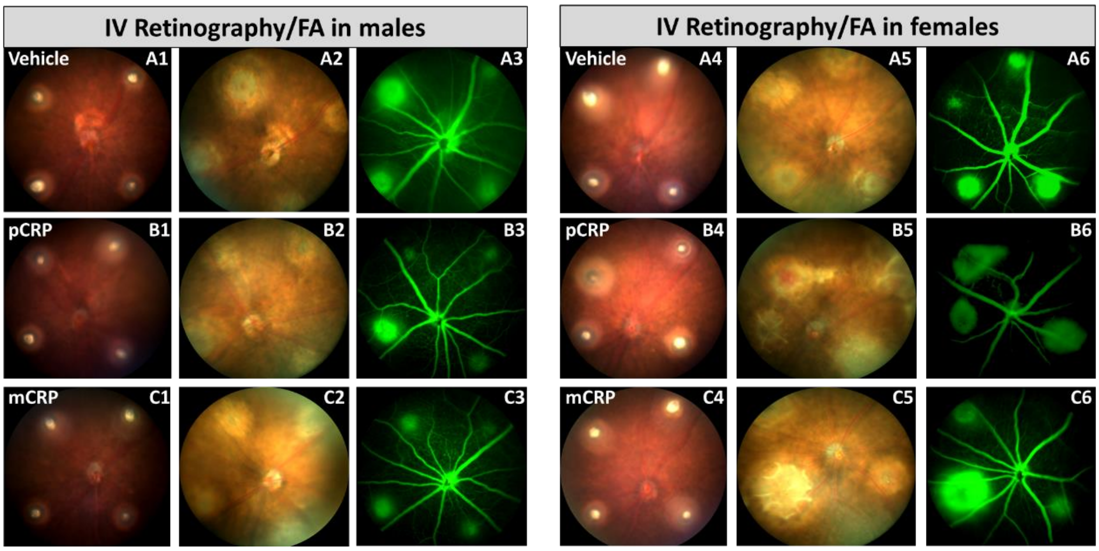

Fig S1. Multimodal imaging of eye fundus in mice IV injected (sacrificed at 9 days postlaser). Retinography immediately after laser induction in males (A1, B1, C1) and females (A4, B4, C4). Retinography after 3 days from laser in males (A2, B2, C2) and females (A5, B5, C5). Fluorescein

angiography (FA) was performed after 3 days from laser in males (A3, B3, C3) and females (A6, B6, C6). IV mice were injected with CRP isoforms 1 day before and 2 days after performing laser. Abbreviations: intravenous=IV, monomeric CRP=mCRP, pentameric CRP=pCRP. Asterisk showed the group statistically significant in the quantification of CNV-lectin areas (Figure 1) quantification.

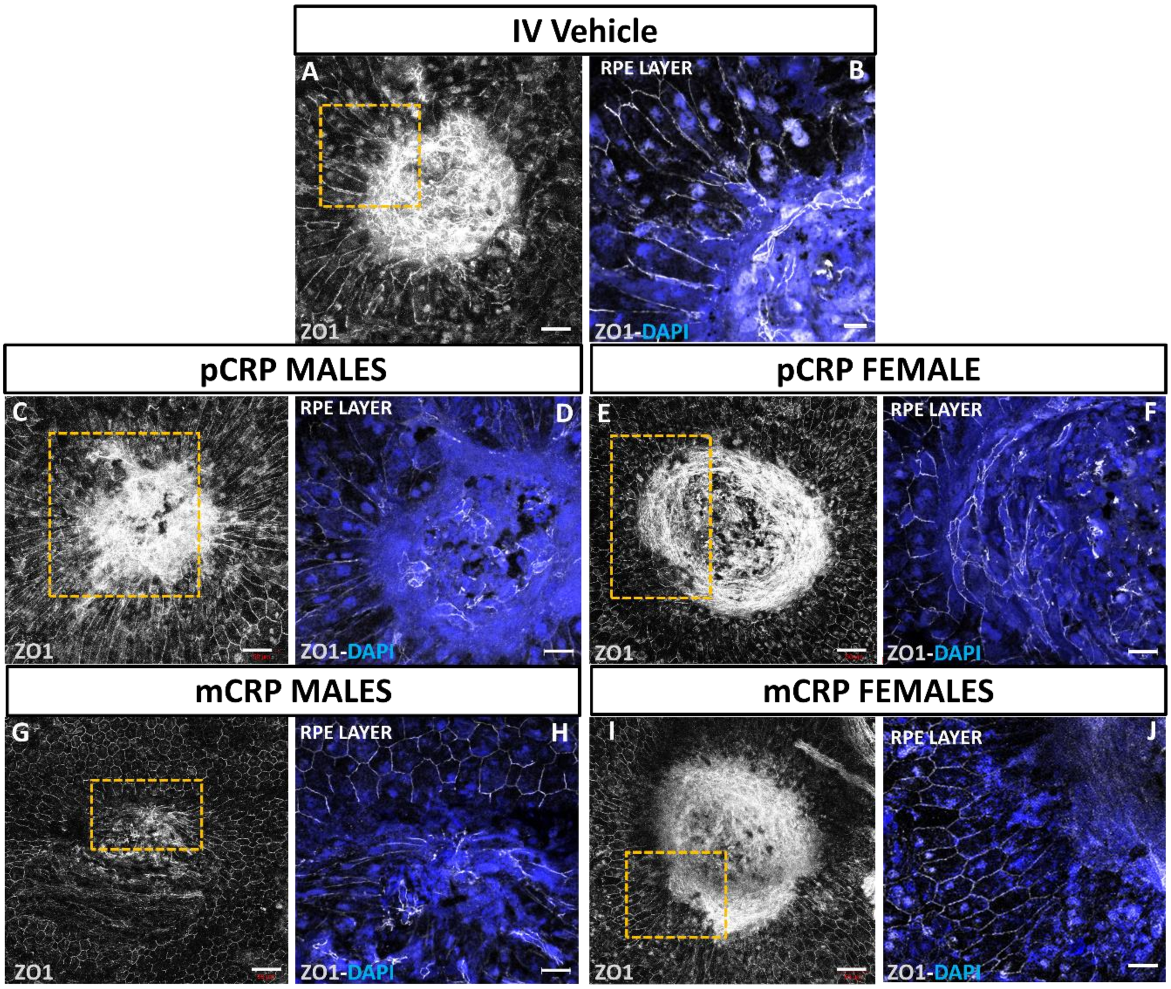

Fig. S2 ZO-1 immunofluorescence in CNV areas in RPE-flatmount after IV injection (sacrificed at 9 days postlaser). In RPE-flatmounts after vehicle (A), we observed ZO-1 staining (white) alteration in detail in RPE layer (B) due to laser induction. After pCRP injection in males (C, D) and females (E, F) similar staining was observed around the angiogenic lesion as we showed in detail in RPE layer (D, F). After mCRP injection in males (G, H) and females (I, J), similar results were observed in ZO-1 staining in detail in males (H) and females (J). DAPI (blue) label nuclei. Scale bar: 50 µm (A, C, E, G, I), 20 µm (B, D, F, H, J). The yellow outlined region indicated the area magnified. IV mice were injected 1 day before and 2 days after performing the laser. Abbreviations: intravenous=IV, mCRP=monomeric CRP, pCRP=pentameric CRP, retinal pigment epithelium=RPE and ZO-1=Zonula occludens-1.

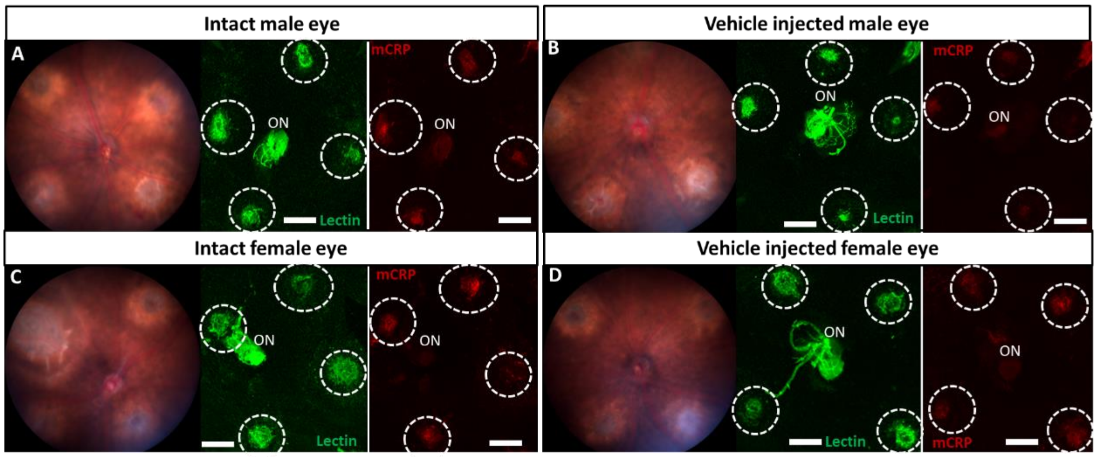

Fig. S3 Retinographies and lectin/mCRP immunofluorescence at one-week postlaser. CNV showed similar immunolabelling for mCRP (red) and lectin staining (green) in males (A, B) and females (C, D) on non-injected eye (A, C) and IVT injection of vehicle (B, D). Scale bar: 100 µm.

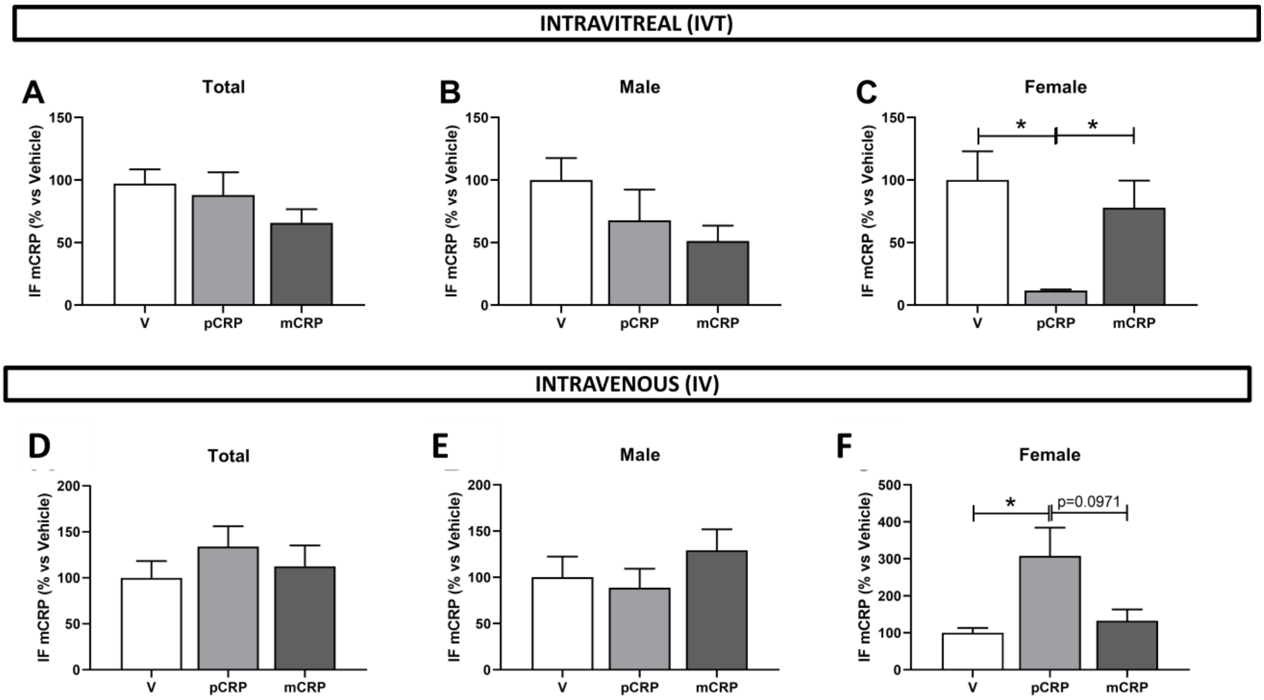

Fig. S4 Percentage of intensity of fluorescence of mCRP (IF-mCRP) after intravitreal (IVT) and intravenous (IV) injection. The quantification was performed in total CNV lesions (A, D) and by gender in males (B, E) and females (C, F). In total samples, IF mCRP were similar in all groups. Males after two isoforms did not show changes in IF mCRP. After IVT injection of mCRP in females IF mCRP was statistically significant increased vs. pCRP (* $p<0.05$) and IVT injection of pCRP showed a statistical decrease vs. vehicle (* $p<0.05$). After IV injection of pCRP in females resulted in an increase of IF mCRP vs. vehicle and almost vs. mCRP ($p=0.071$). Abbreviations: intensity of immunofluorescence mCRP=IF mCRP, intravitreal=IVT, intravenous=IV, monomeric CRP=mCRP and pentameric CRP=pCRP.
